# Supplementary material for: Development and pilot test of a smartphone app for midwifery care in Tanzania: A comparative cross-sectional study
Source: PLoS One. 2023 Mar 31;18(3):e0283808. doi: 10.1371/journal.pone.0283808 (PMC10065243; doi:10.1371/journal.pone.0283808)
Supplement: Attachment — Submitted filename: Responses to the Editor.docx [file pone.0283808.s002.docx]

**Responses to the Editor**

We express our appreciation to the reviewers for their suggestions for revision, which helped us improve our study. We address your comments with point-by-point responses below, including quotations from the manuscript by page and line number. In the manuscript, we denote changes through tracked changes.

**Additional Editor Comments:**

**Comment:** 1) Page 6, line 95 - please move the mHealth definition to the first time the term mHealth is introduced (page 6, line 88)

**Response:** Thank you very much. We moved the definition to the first time the term mHealth is mentioned (page 6, line 86)

**Comment:** 2) Page 9 - please indicate when this study was conducted (what months and years?)

**Response:** We added the study period as “between October 2019 and March 2021.” (page 9, line 139-140)

**Comment:** 3) Page 16, line 269 - Please change "giving a maximum score was 10" to "giving a maximum score of 10"

**Response:** We revised as it was suggested. (page 16, line 264)

**Comment:** 4) Page 24, Line 400-401 - Please change the sentence to read, "To improve the app, they recommended that the reading materials be changed into voice recordings to that they could listen to the contents rather than reading them."

**Response:** We revised as it was suggested. (page 23, line 389-390)

**Comment:** 5) Page 24, line 402 - Please change Swahili to Kiswahili for consistency

**Response:** We revised as it was suggested. (page 24, line 391)

**Comment:** 6) Page 25, line 411 - Please change to "There were no significant differences.."

**Response:** We revised as it was suggested. (page 24, line 400)

Thank you very much again.

We look forward to hearing from you and would be happy to make further changes, if required.
